# Gravidity-dependent associations between interferon response and birth weight in placental malaria

**DOI:** 10.1186/s12936-020-03351-0

**Published:** 2020-08-05

**Authors:** Natalie M. Quanquin, Lauren G. Barres, Saba R. Aliyari, Nathan T. Day, Hoda Gerami, Susan J. Fisher, Abel Kakuru, Moses R. Kamya, Diane V. Havlir, Margaret Feeney, Grant Dorsey, Genhong Cheng, Stephanie L. Gaw

**Affiliations:** 1 Department of Microbiology, Immunology, and Molecular Genetics, University of California, Los Angeles, Los Angeles, CA 90095 USA; 2 Department of Obstetrics & Gynecology, David Geffen School of Medicine At UCLA, Los Angeles, CA 90095 USA; 3 Division of Maternal-Fetal Medicine, Department of Obstetrics, Gynecology & Reproductive Sciences, University of California San Francisco, 513 Parnassus Ave. HSE16, Box 0556, San Francisco, CA 94143 USA; 4 Infectious Diseases Research Collaboration, Kampala, Uganda; 5 Division of Infectious Diseases, Department of Medicine, University of California San Francisco, San Francisco, CA USA; 6 Division of Pediatric Infectious Diseases, Department of Pediatrics, University of California San Francisco, San Francisco, CA USA

**Keywords:** Placental malaria, Interferon, Inflammation, Birth outcomes, Fetal growth restriction, Malaria in pregnancy, Gravidity, Birth weight

## Abstract

**Background:**

Maternal malarial infection leads to poor perinatal outcomes, including low birth weight from preterm delivery and/or fetal growth restriction, particularly in primigravidas. In placental malaria, *Plasmodium falciparum*-infected red blood cells cause an inflammatory response that can interfere with maternal–fetal exchange, leading to poor growth. The type I interferon (IFN-I) pathway plays an immunomodulatory role in viral and bacterial infections, usually by suppressing inflammatory responses. However, its role in placental malaria is unknown. This study examines the cytokine responses in placental tissue from subsets of malaria-infected and uninfected women, and attempts to correlate them with particular birth outcomes.

**Methods:**

40 whole placental biopsy samples were obtained from pregnant women at least 16 years of age recruited to a larger prospective chemoprevention trial against malaria. These were patients at Tororo District Hospital in Uganda, an area of high malaria endemicity where approximately 40% of women have evidence of malaria infection at delivery. They were regularly followed at a local clinic and monitored for fever, with blood smears performed then and at time of delivery to diagnose malaria infection. Placenta biopsies were taken for histological diagnosis of placental malaria, as well as quantitative PCR analysis of genes in the IFN-I pathway (IFN-β, IL-10 and MX-1). Parameters such as infant birth weight and gestational age were also recorded.

**Results:**

Histological analysis revealed placental malaria in 18 samples, while 22 were found to be uninfected. RT-PCR analysis showed a four-fold increase in IFN-β and IL-10 expression in multigravidas with placental malaria when compared to gravidity-matched, uninfected controls. This effect was not observed in primigravidas. Interestingly, linear regression analysis showed a positive association between IFN-β levels and higher birth weights (β = 101.2 g per log2-fold increase in IFN-β expression, p = 0.042). This association was strongest in primigravidas with placental malaria (β = 339.0, p = 0.006).

**Conclusions:**

These results demonstrate differential regulation of the IFN-I pathway in placental malaria according to gravidity, with the greatest anti-inflammatory response seen in multigravidas. The association between IFN-β levels and higher birth weight also suggests a protective role for IFN-I against fetal growth restriction in placental malaria.

## Background

Almost half of the world’s population is at risk for malarial infection, which led to 429,000 deaths in 2015 [1]. Perhaps less well known is the toll this infection takes during pregnancy, where it has been associated with poor perinatal outcomes, including low birth weight and death [2–5]. The body’s natural immune response to malaria involves antibody-mediated protection and innate immune responses mediated by cytokines and downstream genes activated by signaling pathways. These innate reactions can be either beneficial or harmful to the host, depending on the malaria parasite stage and the infected organ [6–10]. In placental malaria, *Plasmodium falciparum-*infected red blood cells (RBCs) cause an inflammatory response that can interfere with maternal–fetal interchange and lead to fetal growth restriction [11–16].

Malaria is caused by the protozoan parasite *Plasmodium*, spread by the bite of infected *Anopheles* mosquitoes. Five species are known to infect humans and are endemic to particular regions across the globe [17]. *Plasmodium falciparum* is considered the most pathogenic, causing systemic infection that can be fatal when spread to the brain. During pregnancy, *P. falciparum* can also sequester infected RBCs in the placental intervillous spaces [6, 18]. In 2015, it was estimated that 28 million pregnant women in sub-Saharan Africa were at risk of malaria [1], with the median prevalence of placental malaria in all pregnant women estimated to be 26–28% [11, 12]. Studies have found only a low frequency of the parasite crossing into the fetal circulation to cause congenital infection, possibly due to structural barriers to infected RBCs, or host protection by maternal antibodies [18, 19]. However, its presence in the placental circulation or tissues does impair the critical exchange of nutrients and oxygen with the fetus [11]. This group and others have found that a high systemic malaria burden during pregnancy leads to an increased likelihood of placental malaria, which is also associated with an increased risk of adverse birth outcomes [2, 5, 13–15, 20]. It is unclear if this is a response to virulence factors on the surface of infected RBCs, or due to inflammatory by-products that accumulate after RBC invasion by the parasite [11–16].

The growth of the developing fetus is dependent on the efficient delivery of maternal blood to the placenta. While *Plasmodium* parasites do not invade placental cells and therefore do not directly damage the integrity of this interface, they do trigger inflammatory changes that impair trophoblast migration into the placenta, which is necessary for maternal spiral arteries to transform and improve the blood supply [11, 16]. This vascular insufficiency can lead to fetal growth restriction [16], causing adverse infant outcomes such as preterm birth, low birth weight, and being small for gestational age [7, 21]. Up to 20% of all low birth weight deliveries in Africa have been attributed to malaria in pregnancy, causing 75,000–200,000 infant deaths annually [11, 22]. Malaria has likewise been associated with approximately 36% of all preterm births in endemic regions [12].

There have been numerous efforts made towards elucidating the nature of the immune response to malaria. It is believed that adaptive immunity plays a significant role in protecting the host from recurrent malarial infections, particularly in placental malaria, where subsequent pregnancies appear to show a reduced risk of infection and clinical sequelae compared to primagravida cases [7, 23, 24]. By contrast, the innate immune system has been shown to be critical in helping to clear the host's parasite burden [6–10, 25, 26]. Some studies point to the protective role of type I interferons α and β (IFN-I), cytokines that have been linked to the initiation of cell-mediated responses important for protection against viral infections [8–10, 25]. However, IFN-I also has potent anti-inflammatory properties that can be detrimental during chronic infection [26]. Against malaria, IFN-I has been linked to both protective and deleterious effects, depending on the stage and site of infection [8–10, 25]. In placental malaria, this relationship is further complicated by innate responses from both the fetus and maternal compartments that may act in opposition [6, 26]. Type II interferon (IFN-γ) promotes antigen presentation and directly activates antiviral mediators and macrophages, resulting in a pro-inflammatory phenotype. Inflammatory cytokines such as IL-12, TNF and IL-6, are then kept in balance by the anti-inflammatory effects of IL-10 and other downstream effectors of IFN-I [6, 8, 26].

This study sought to compare the cytokine expression in placental samples with histologic evidence of malaria infection versus uninfected tissue, to see if it corresponded to either an increased or suppressed inflammatory pattern. Whether this phenotype changed based on gravidity, an important risk factor for worse malaria outcomes in pregnancy, was also examined. Placental tissue was collected from 40 patients recruited into a large malaria chemoprevention trial at the Tororo District Hospital in Uganda [27], an area of high endemicity where approximately 40% of women have evidence of malaria infection at delivery [28]. Their infant clinical outcomes were included in this study, and a relationship was observed between the expression of these cytokines and the incidence of preterm labour and birth weight.

## Methods

### Study design, site and population

This is a nested prospective cohort of 300 pregnant women enrolled in a randomized controlled trial (RCT) in Tororo, Uganda for one of three chemoprophylactic regimens against malaria [27]. Tororo is a rural district in southeastern Uganda with an entomologic inoculation rate estimated at 310 infectious bites per person year in 2012 [28]. The RCT was a three-arm, double-blinded, placebo-controlled trial of sulfadoxine-pyrimethamine (SP) given every 8 weeks, dihydroartemisinin-piperaquine (DP) given every 8 weeks, or DP given every 4 weeks for intermittent preventative treatment in pregnancy (IPTp). Patients were at least 16 years of age at enrollment, and obstetrically dated with an estimated fetal gestational age of 12–20 weeks confirmed by ultrasound prior to enrollment. Patients were followed regularly at the local study clinic and blood smears were performed after any fever and at the time of delivery to diagnose malaria. Placenta tissue biopsies were taken for histological diagnosis of placental malaria. Parameters such as birth weight and gestational age were also recorded, and patients were catagorized as “small for gestational age” if they charted below the tenth percentile based on growth curve standards for East African children [29].

Additional placenta biopsy specimens taken from all available RCT enrollees delivering from January to February 2015 were selected for this study for RT-PCR analysis.

### Sample collection, histology and quantitative RT-PCR

Whole placenta specimens were obtained within 30 min of delivery. As part of the parent study, 1 × 1 cm blocks were collected, fixed and processed as previously described [2] for histological diagnosis of placental malaria according to Rogerson criteria [30, 31]. For this analysis, separate full-thickness placental biopsy samples measuring 1 × 1 cm were excised, washed in phosphate-buffered saline, and preserved in RNAlater per manufacturer instructions (Qiagen; Germantown, MD, USA). Tissue samples were frozen at −80 °C for long term storage and shipped on dry ice to UCLA. On arrival, samples were thawed and broken apart by sonication, then resuspended in TRIzol reagent (Thermo Fisher Scientific; Waltham, MA, USA) for RNA isolation by standard isopropanol precipitation and RNeasy Tissue Kit (Qiagen; Germantown, MD, USA). RNA was quantified and 1 μg was reversed transcribed into cDNA using iScript (Bio-Rad; Hercules, CA, USA) according to the manufacturer's instructions with random hexamer primers. qRT-PCR analysis was done using the iCycler thermocycler (Bio-Rad) in a final volume of 20 μl, with amplification conditions of 95 °C (3 min), 40 cycles of 95 °C (20 s), 55 °C (30 s), 72 °C (20 s). Primers for IFN-β, MX-1, IL-10, IFN-γ, IL-6, and TNF were described previously [32]. Ct values were normalized against the housekeeping gene H3B64 and fold induction was normalized to the untreated control.

### Statistical analysis

When comparing patient characteristics, P-values were calculated by two-tailed student’s t-tests or Fisher’s exact test. For comparisons of cytokine expression between uninfected and placental malaria cases, P-values were calculated by two-tailed student’s t-test with Bonferroni’s correction. Associations with gene expression levels and birth weight were analysed by linear regression. All statistical analysis was performed with Stata 13 or Prism 6.

## Results

### Associations of placental malaria infection with adverse outcomes

This study analysed placental tissue from a subgroup of 300 women enrolled in a chemoprophylactic trial against malaria, including all samples from the 40 who delivered between January and February of 2015. Those 40 patients were evenly distributed between the three treatment arms. Through histopathological examination, placental malaria was diagnosed in 18 samples (45%), and the remaining 22 placentas (55%) were characterized as “uninfected”. In all 18 cases of placental malaria, no parasites were visualized–only pigment was found as evidence of past infection. Active infection diagnosed by tissue histology was also rare in the larger cohort. All patients were provided with a long-lasting insecticidal net at the time of enrollment into the Uganda study, where compliance was noted to be >85% between all treatment groups. Patients were asked to report to the study clinic for any fevers, at which time blood smears were taken to evaluate for malaria infection. Interestingly, of the 22 women in the “uninfected placenta” group, 4 (18%) actually had symptomatic malaria during pregnancy. Furthermore, of the 18 women with placental malaria, only 8 (44%) had symptomatic malaria diagnosed during pregnancy. The remaining characteristics of these two groups are outlined in Table 1.

**Table 1:** Patient characteristics and birth outcomes

	Uninfected placenta (n = 22)	Placental malaria (n = 18)	*p*
Maternal age (years)	23.6 ± 4.0	19.0 ± 1.3	< 0.001
Gestational age (weeks)	39.9 ± 1.4	39.3 ± 2.5	0.362
Malaria in pregnancy	4 (18%)	8 (44%)	0.093
Gravidity			0.003
Primigravida	4 (18%)	12 (67%)	
Multigravida	18 (82%)	6 (33%)	
Preterm labour (<37 w)	0 (0%)	2 (11%)	0.196
Cesarean Delivery	3 (13%)	1 (6%)	0.613
Birth weight (grams)	2988 ± 340	2950 ± 642	0.811
Low birth weight (<2500 g)	1 (5%)	3 (17%)	0.310
Small for gestational age (<10% expected birth weight)	4 (18%)	5 (28%)	0.705

Data represent the mean and standard deviation or n (%). P values were calculated by two-tailed student’s t-tests or Fisher’s exact test.

### Anti-inflammatory cytokines are upregulated in multigravidas with placental malaria

RNA extracted from whole placental biopsies was analysed by quantitative RT-PCR (qRT-PCR) for expression of six cytokines of interest in the type I (IFN-β, MX-1, and IL-10) and type II (IFN-γ, IL-6, and TNF) interferon pathways. This qRT-PCR analysis was performed in a blinded fashion (with no knowledge of the placental malaria status or clinical outcomes in the generation of the expression data). The results were then stratified according to whether the samples were “uninfected” or diagnosed with placental malaria, as well as maternal gravidity status.

**Fig. 1:**
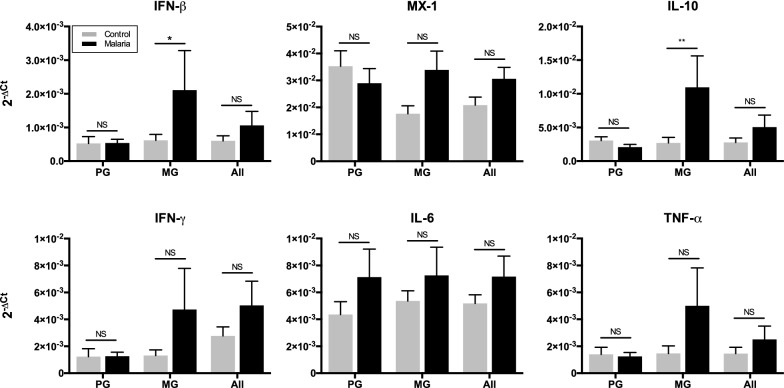
Gravidity-dependent changes in the expression of interferon pathway genes in placental malaria. Quantitative RT-PCR was performed on RNA purified from whole placental biopsies obtained within 30 min of delivery. Expression of IFN-β, MX-1, IL-10, IFN-γ, IL-6 and TNF genes is shown. Ct values were normalized against the housekeeping gene GAPDH. Data are presented as the mean ± SEM of 2-ΔCt for each group. P-values were calculated by two-tailed student’s t-test with Bonferroni’s correction, comparing uninfected controls vs. placental malaria cases. Abbreviations:* PG* primigravida,* MG* multigravida,* NS* not significant; * p < 0.05, ** p < 0.01

In multigravida samples, placental malaria was associated with elevated transcription levels of all six cytokines compared to uninfected controls (Fig. 1). This difference was only significant for IFN-β and IL-10, where up to a four-fold increase in gene expression was observed. Interestingly, these results were not seen in primigravida samples, where malaria infection did not appear to induce any change in the transcript levels of five of the six selected cytokines. The expression of IL-6 in placental malaria samples was slightly elevated in both multigravida and primigravida samples when compared to gravidity-matched, uninfected controls, however the differences were not significant.

### IFN-β expression in placental malaria is associated with higher birth weights

**Fig. 2:**
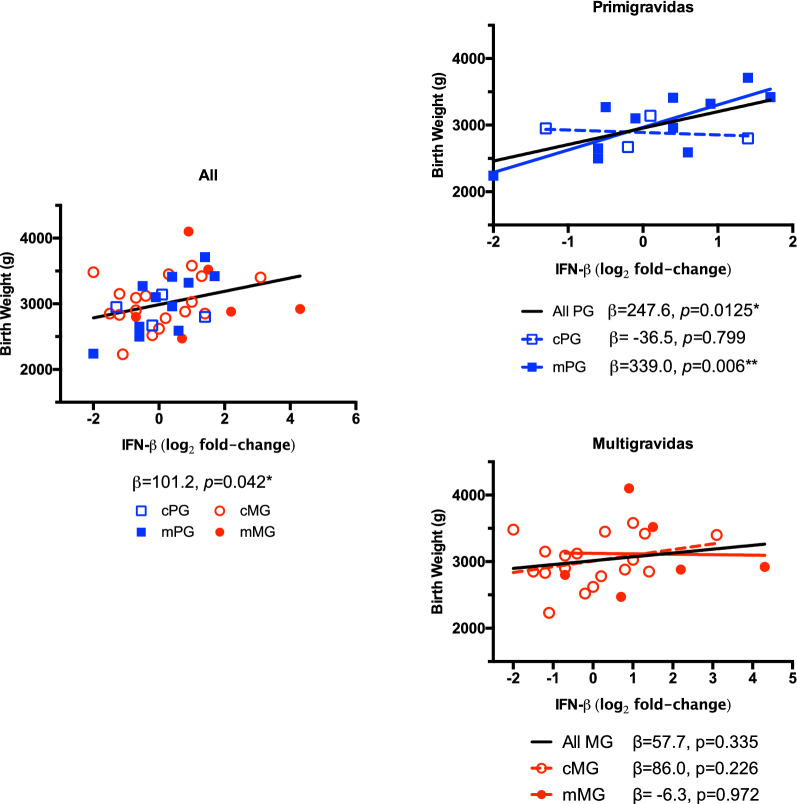
Placental expression of IFN-β in relation to birth weight. Relative expression of IFN-β was calculated and presented as a log2 fold-change, compared to the mean of gravidity-matched controls. Abbreviations:* cPG* control/primigravida, open blue squares;* mPG*, malaria/primigravida, closed blue squares;* cMG* control/multigravida, open red circles;* mMG* malaria/multigravida, closed red circles; * p < 0.05, ** p < 0.01, calculated by linear regression

The cytokine expression profiles of individual samples were plotted with the corresponding infant birth weight to look for possible relationships between the inflammatory response and clinical outcomes. Linear regression analysis of all samples combined showed a positive association between IFN-β levels and higher birth weights (Fig. 2; top panel, β = 101.2 g per log2-fold increase in IFN-β expression, p = 0.042). When looking at individual groups, the association was strongest in primigravidas with placental malaria, where each log2-fold increase in IFN-β expression was associated with an over 300 g increase in birth weight (solid blue squares, β = 339.0, p = 0.006). This association between IFN-β expression levels and birth weight was not observed in multigravidas.

## Discussion

This study describes the differential regulation of the IFN-I pathway in placentas affected by placental malaria according to gravidity. Specifically, a four-fold increase in IFN-β and IL-10 expression was found in multigravidas with placental malaria when compared to gravidity-matched, uninfected controls, which was absent in primigravidas. There was also a positive association between IFN-β levels and higher birth weights, especially in primigravidas with placental malaria (β = 339.0, p = 0.006), the demographic group at highest risk of adverse outcomes from placental malaria.

Studies have shown primigravidas to be more susceptible than multigravidas to complications during malaria infection, such as low birth weight from preterm delivery and/or fetal growth restriction [2, 7, 23, 24]. One possibility is that primigravidas tend to be younger women, who would have less lifetime exposure and immune memory to the parasite than multigravidas. Indeed, placental malaria was found to be associated with primigravidity and younger maternal age in this patient cohort. Of the 40 women, 18 were diagnosed with placental malaria, of which 12 (67%) were primigravid. Likewise, of the 22 uninfected cases, 18 (82%) were multigravid. In addition, the mean age of women with placental malaria in this study was 19 ± 1.3 years, compared to 23.6 ± 4.0 years for uninfected women. These were the only patient characteristics that were found to be significantly different between the placental malaria and uninfected placenta groups in this study. However, it was noted that infants born to the placental malaria group had a higher incidence of low birth weight and being small for gestational age. Although those differences were not found to be significant in this small cohort, a separate analysis published on all 282 patients in the RCT that had placental pathology and birth outcome data available showed a significant association between placental malaria and preterm birth [aRR 5.64 (1.46–21.8)], with a trend towards increased risk for low birth weight and being small for gestational age [2].

The placenta represents a complex environment where the maternal immune defenses are intentionally suppressed to prevent rejection of the fetus. However, malaria infection of the placenta stimulates cell-mediated immune responses that trigger the local production of inflammatory cytokines such as IFN-γ, IL-2, and TNF, which are believed to account for the placental pathology and adverse pregnancy outcomes that are particularly noted in primigravida women [33–35]. Pathogen-associated molecular patterns on malaria parasites activate innate immune signaling pathways in the host, including both the type I interferon (such as IFN-β) and type II interferon (IFN-γ) responses [36]. The latter triggers the rapid induction of pro-inflammatory cytokines, chemokines, and growth factors that initiate tissue regeneration and host defenses against infection. IFN-γ and IL-12 play crucial roles in the clearance of intracellular pathogens including malaria, with IFN-γ responses shown to be associated with protection from *P. falciparum* [37], and low IL-12 levels being observed in severe malaria infections [38]. Unfortunately, over-activation of these pathways can also lead to cytokine storms and excessive inflammation, leading to tissue damage. Therefore, anti-inflammatory cytokines such as IL-10 induced by IFN-β serve as a negative feedback mechanism to keep this system in check [8]. IL-10 has been shown to be critical in curbing mortality from infection, and its absence in knockout mice leads to increased susceptibility to inflammatory and autoimmune disorders [39–41]. IL-10 also strongly inhibits the production of cytokines such as TNF, IL-I and IL-6, which are involved in malaria pathology [42]. The results did show an increase in IFN-γ, IL-6, and TNF expression in placental malaria versus uninfected samples from multiparous women, although it did not reach a level of statistical significance. In primiparous women, this increase was only seen for IL-6. However, this study looked specifically at expression in placental tissue, whereas most others report on expression in peripheral blood, and do not stratify based on gravidity.

When examining the transcription levels of a subset of cytokines in the IFN-I signaling pathway, a four-fold increase was found in IFN-β and IL-10 expression in multigravidas with placental malaria when compared to gravidity-matched, uninfected controls. Interestingly, this pattern was not seen in primigravid samples. As cases of placental malaria have better perinatal outcomes in multigravida pregnancies than in primigravida pregnancies, and placental inflammation is a mechanism for fetal growth restriction, it was hypothesized that the increased expression of these cytokines in multigravid women is responsible for this difference. Further supporting this finding, linear regression analysis of the cohort data showed a positive association between IFN-β levels in placental samples and higher birth weights in the corresponding infants. Of note, this association was strongest in primigravidas with placental malaria. One possibility is that their overall lower expression of IFN-β makes them more sensitive to its effects. The mechanism by which increased gravidity raises the expression of these cytokines in placental malaria remains unclear. However, these results demonstrate a differential regulation of the IFN-I pathway in placental malaria according to gravidity, with the greatest anti-inflammatory response seen in multigravidas. This association between IFN-β levels and higher birth weight may point to a protective role for IFN-I against fetal growth restriction in placental malaria.

## Conclusion

In placental malaria, the type I interferon pathway is differentially regulated in primigravidas compared to multigravidas. Upregulation of the type I interferon pathway in multigravid women suggests a gravidity-dependent anti-inflammatory response. The association between IFN-β levels and higher birth weight suggests a protective role for type I interferons against fetal growth restriction in placental malaria.

## Data Availability

The patient characterstics and cytokine expression data used to support the findings of this study are included within the article.

## Abbreviations

IFN-I: Type I interferon
IFN-α: Interferon alpha
IFN-β: Interferon beta
IL-10: Interleukin 10
MX-1: Myxovirus resistance 1
RBC: Red blood cell
IFN-γ: Interferon gamma, also known as the Type II interferon
IL-12: Interleukin 12
TNF: Tumour necrosis factor
IL-6: Interleukin 6
RCT: Randomized controlled trial, herein refering to the parent study in [21]
SP: Sulfadoxine-pyrimethamine
DP: Dihydroartemisinin-piperaquine
qRT-PCR: Quantitative reverse transcription polymerase chain reaction
